# Enhanced Light Extraction from Bottom Emission OLEDs by High Refractive Index Nanoparticle Scattering Layer

**DOI:** 10.3390/nano9091241

**Published:** 2019-08-31

**Authors:** Chan Young Park, Byoungdeog Choi

**Affiliations:** Department of Electrical and Computer Engineering, Sungkyunkwan University, 2066 Seobu-Ro, Jangan-Gu, Suwon, Gyeonggi-do 16419, Korea

**Keywords:** OLED, light extraction, outcoupling, nanoparticle, high refractive index, scatter, efficiency, display, YSZ

## Abstract

High refractive index nanoparticle material was applied as a scattering layer on the inner side of a glass substrate of a bottom emission organic light emitting diode (OLED) device to enhance light extraction and to improve angular color shift. TiO_2_ and YSZ (Yttria Stabilized Zirconia; Y_2_O_3_-ZrO_2_) were examined as the high refractive index nanoparticles. The nanoparticle material was formed as a scattering layer on a glass substrate by a coating method, which is generally used in the commercial display manufacturing process. Additionally, a planarization layer was coated on the scattering layer with the same method. The implemented nanoparticle material and planarization material endured, without deformation, the subsequent thermal annealing process, which was carried out at temperature ranged to 580 °C. We demonstrated a practical and highly efficient OLED device using the conventional display manufacturing process by implementing the YSZ nanoparticle. We obtained a 38% enhanced luminance of the OLED device and a decreased angular color change compared to a conventional OLED device.

## 1. Introduction

In flat-panel display devices, organic light emitting diodes (OLEDs) are widely used, from mobile phones to large size TVs because OLED has excellent electro-optical properties for display products compared to widely used liquid crystal displays (LCDs). Among the properties, the efficiency of OLED devices is an important factor for practical OLED display products. 

There are many studies on enhancing the efficiency of OLEDs in various technology fields, such as materials, device structures, fabrication processes, circuit design, and circuit driving. The efficiency of OLEDs has been enhanced by improving the characteristics of organic materials comprised in OLED devices and by optimizing the structures of OLED devices [1,2,3,4]. However, other approaches for finding solutions for the improvement of the outer structures of OLED devices have been studied in order to enhance the efficiency of OLED devices [5,6]. 

**Figure 1:**
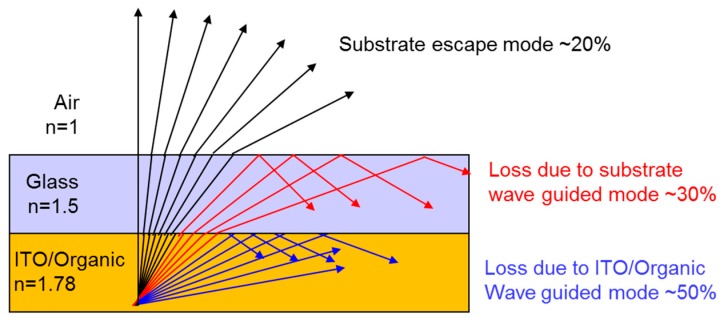
Schematic diagram of multi-layer OLED structure and optical ray diagram of light propagation via various modes, i.e., substrate escape, substrate wave guided mode, and ITO/Organic wave guided mode.

As shown in Figure 1, 20% of emitted light is extracted outside of OLED devices; however, 80% of emitted light is trapped inside OLED devices and dissipated, which is caused by the difference of the refractive index of each layer comprised in OLED devices [5,7]. Various ways were tried to extract the trapped light from the OLED devices [5,6,7]. Several methods have been proposed to enhance the light extraction from OLEDs, including mesh structures on the glass substrate [8], silica micro spheres [9], scattering layers [10], embedded low-index grids [11], photonic crystals [12,13], micro pyramids [14], and micro lens arrays [15,16,17,18]. However, these methods could be used for limited applications such as illumination products without pixels. Most methods for enhancing the efficiency of OLED are implemented on the outer side of the glass substrate, where the distance between the light source and the emitting structures is relatively long, due to the thickness of the glass substrate. In display products with many pixels, these methods cause image blurring, which is a mixture of light from adjacent pixels and poor image quality by a diffusion effect. Other approaches have been studied to enhance the light extraction of OLEDs, adopting nano sized structures to OLEDs. By the scattering effect of the nano structures, such as gratings [19,20], metallic nanoparticles [21,22,23,24,25], and buckled substrates [24,26,27], the light trapped in glass substrate can escape. However, nano structures have limited application in OLEDs due to the complicated process, requiring high cost. Additionally, their size limitation impedes their implementation in practical OLED products. Hence, the nanoparticles fabricated by easy methods or by methods compatible with conventional manufacturing processes suitable for large areas are more beneficial for practical OLEDs devices.

In this study, we applied high refractive index nanoparticle material as a scattering layer on the inner side of the glass substrate of the bottom emission OLED device to enhance light extraction and to improve angular color shift. The nanoparticle scattering layer is located near the emitting layer of the OLED device, compared to previous approaches mentioned above, so it rarely affects the image quality of the OLED display. We demonstrated a practical highly efficient OLED device using the conventional display manufacturing process by implementing the YSZ nanoparticle as the high refractive index scattering layer. We obtained a 38% enhancement of luminance of the OLED device and a white angular dependency (WAD) of 0.005 at 60 degrees from normal direction.

## 2. Materials and Methods 

TiO_2_ and YSZ (Yttria Stabilized Zirconia; Y_2_O_3_-ZrO_2_) were investigated as high refractive index nanoparticle materials for scattering layer. The refractive index of TiO_2_ nanoparticles is 2.5 and that of YSZ is 2.13, respectively. Both TiO_2_ and YSZ nanoparticles are suitable for high refractive index materials compared to that of glass substrate. The size of the TiO_2_ nanoparticle was 250–300 nm, while the size of the YSZ nanoparticle was 100–150 nm. The nanoparticles were dispersed in propylene glycol methyl ether acetate (PGMA) solvent for applying to the slit coating method, which is used in the conventional display manufacturing process for large areas. The nanoparticle suspension was coated on the glass substrate, forming the high refractive index scattering layer, followed by a thermal curing process at 300–500 °C in a furnace for 30 min. The curing temperature can be controlled according to the state of the coating layer, without any deformation. A SiO_2_ solution, which was normally used at spin-on glass (SOG), was used as a planarization material, which was coated on the nanoparticle scattering layer. The refractive index of the SiO_2_ planarization layer was 1.45, which was similar to that of the glass substrate. The planarization layer was formed on the scattering layer with the same method as the scattering layer. It was cured at 500 °C in a furnace for 30 min. The planarization layer had a flat surface covering the roughness of the scattering layer caused by the distribution of nanoparticles. Therefore, subsequent manufacturing process can be performed easily, like on the glass substrate surface. Next, the conventional low temperature poly silicon (LTPS) thin film transistor (TFT) manufacturing process was performed on the planarization layer. In the LTPS TFT manufacturing process, the thermal annealing process is required at the highest temperature, approaching 580 °C, which can cause deformation of the nanoparticle scattering layer and the planarization layer. The proposed materials should endure the high temperature during LTPS TFT manufacturing process without any deformation.

**Figure 2:**
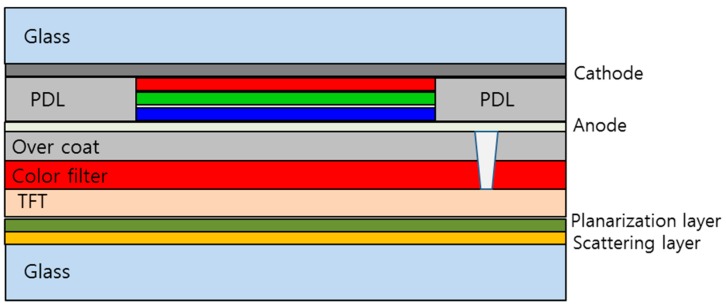
Schematic diagram of the bottom emission white OLED device with the scattering layer on the inner side of glass substrate.

A conventional bottom emission white OLED display device was used to investigate the effect of the high refractive index nanoparticle scattering layer, as represented in Figure 2. On the glass substrate, the nanoparticle scattering layer, the planarization layer, and the TFT layer were fabricated sequentially. Next, a color filter and an over coat layer were formed to reproduce color images on the OLED display device. On the indium tin oxide (ITO) anode electrode, the white OLED device, comprised of blue, green, and red emitting layers stacked up, were deposited by a thermal evaporation process. The device was finalized with an aluminum cathode and a top glass substrate. The size of the glass substrate was 370 × 470 mm. Additionally, an OLED device without a nanoparticle scattering layer and a planarization layer was also fabricated as a reference device.

Measurements of OLED properties were performed by recording current–voltage characteristics as well as electro-luminescence (EL) spectra. The current–voltage was acquired using a Keithley 236 voltage source unit, while the EL intensity and spectral characteristics of the devices were measured with a calibrated silicon photodiode (Hamamatsu Photonics, Hamamatsu, Japan, S5227-1010BQ), a photomultiplier tube, and a spectroradiometer (Minolta, Osaka, Japan, CS-1000).

## 3. Results and Discussion

### 3.1. Properties of the Nanoparticle Scattering Layer

The scattering layer with embedded TiO_2_ nanoparticles was fabricated using the conventional slit coating method. However, TiO_2_ nanoparticles are hard to disperse in PGMA solvent, therefore they were not dispersed uniformly. The coating thickness of the TiO_2_ nanoparticle scattering layer was 1.5 μm, then 1 μm thick SiO_2_ was coated as the planarization layer. Table 1 shows the optical properties of the TiO_2_ nanoparticle scattering layer, such as transmittance and haze. We observed 70% of total transmittance at 550 nm wavelength and 55–82% of haze. The surface of the TiO_2_ nanoparticle layer was rough, even though the planarization layer was applied on it. Considering its surface roughness, it would cause poor characteristics in the subsequent LTPS TFT process. Due to poor dispersivity of TiO_2_ nanoparticles in the PGMA solvent, the curing temperature was limited to 300 °C, which was lower than the annealing process temperature of 580 °C in the subsequent LTPS TFT process. Despite the high refractive index of TiO_2_ nanoparticles, the use of conditions such as particle size, sorts of solvent, and better dispersion of TiO_2_ nanoparticles is required.

**Figure 3:**
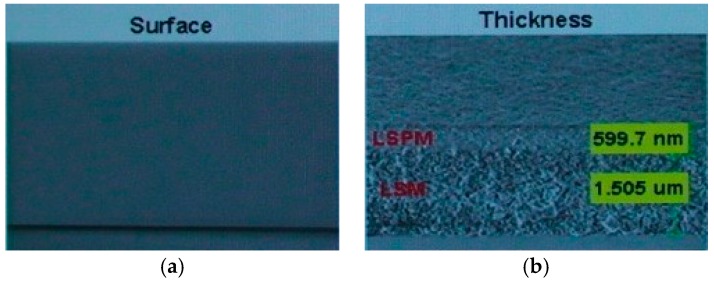
SEM images of the YSZ nanoparticle scattering layer (LSM) and the planarization layer (LSPM) after the curing process: (**a**) Top view of planarization layer; (**b**) Cross sectional view of the scattering layer and the planarization layer.

On the other hand, the scattering layer with embedded YSZ nanoparticles was successfully fabricated using the conventional slit coating method. ZrO_2,_ comprising YSZ has a monoclinic crystal structure at room temperature. It changes to a tetragonal crystal structure and a cubic crystal structure as temperature increases. YSZ comprised of Yttria doped ZrO_2_ has a stable crystal structure even when temperature increases. YSZ has characteristics of chemical stability, thermal resistance, low thermal conductivity, and high strength. It has similar optical properties to ZrO_2_, including a high refractive index. The YSZ nanoparticle scattering layer was coated with various thicknesses of 1.5 μm, 2.5 μm, 4.0 μm, and 7.0 μm, respectively, and then was cured at 500 °C. Next, 1 μm of SiO_2_ was coated as the planarization layer. Figure 3 illustrates the morphology of the YSZ nanoparticle scattering layer and the planarization layer including a cross sectional view, measured by scanning electron microscopy (SEM). The surface of the planarization layer was flat with small defects, like the glass substrate. Moreover, the density of the YSZ nanoparticle scattering layer was lower than that of the planarization layer. The change of thickness of the planarization layer from 1 μm at the coating step to 0.6 μm after the curing step means that the coated SiO_2_ solution permeated into the vacancies of the nanoparticle scattering layer. The large difference between refractive index of the YSZ nanoparticles (2.13) and the SiO_2_ material (1.45) within the interpenetrating areas enhanced the scattering effect. Table 2 shows optical properties of the YSZ nanoparticle scattering layer. We obtained 58–78% of total transmittance of the YSZ nanoparticle scattering layer at 550 nm wavelength and 24–55% of haze. It was observed that transmittance decreased and haze increased as the thickness of scattering layer was increased. This means that the thick scattering layer enhances the scatter effect due to increasing light diffusion during its propagation. Therefore, the scatter effect and transmittance can be controlled by modulating the thickness of the scattering layer. Moreover, the YSZ nanoparticle scattering layer and the planarization layer have a uniform surface and a high curing temperature of 500 °C, so the subsequent LTPS TFT process can be performed easily.

### 3.2. Performance of the OLED Device with High Refractive Index Nanoparticle Scattering Layer

**Figure 4:**
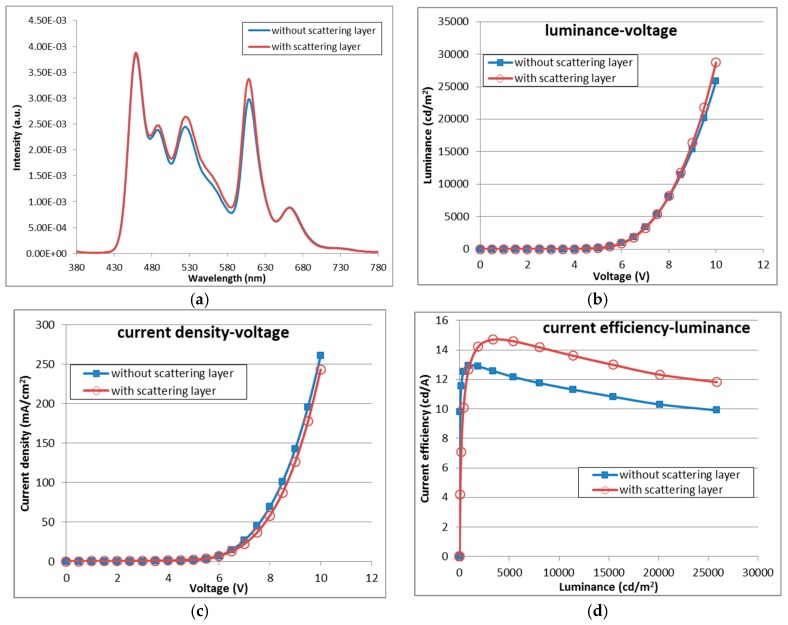
Characteristics of OLED devices with and without the YSZ scattering layer. The thickness of the YSZ scattering layer is 2.5 μm: (**a**) Electroluminescence spectrum of the OLED devices. The spectrum data are normalized at 1mA/cm^2^; (**b**) Characteristics of luminance for different voltages; (**c**) Characteristics of current density for different voltages; and (**d**) Characteristics of current efficiency for difference luminance.

A conventional bottom emission white OLED device for flat-panel display products was fabricated. The characteristics of the OLED devices with and without the YSZ scattering layer are illustrated as Figure 4. The OLED devices had three peaks at red, green, and blue color wavelengths. The OLED devices were used as the light sources to investigate the enhancement of light efficiency of the OLED with an embedded YSZ scattering layer.

In the previous study, employing a scattering layer to OLED devices as a layer or film coated on outer surface of the glass substrate reported enhanced light efficiency of OLEDs [10]. However, the distance from the light emitting layer to the scattering layer was very long, including the thickness of the glass substrate 0.5 mm compared to the pixel pitch 80 μm. Therefore, the light scatter effect caused mixing colors among pixels and led to image blur on the display screen. In this work, we implemented the scattering layer on the inner surface of the glass substrate. The distance from the light emitting layer to the scattering layer was short, about half of the pixel pitch, so a good image quality without image blur could be achieved by the proposed method. The emitted light at the OLED device between the anode electrode and the cathode electrode propagated to various directions while it passed the scattering layer. The scattered light changed the direction of propagation through the glass substrate, which limited the total internal reflection. Therefore, the light from the OLED substrate wave guided mode could be extracted, which enhanced the light efficiency of the OLED device. 

**Figure 5:**
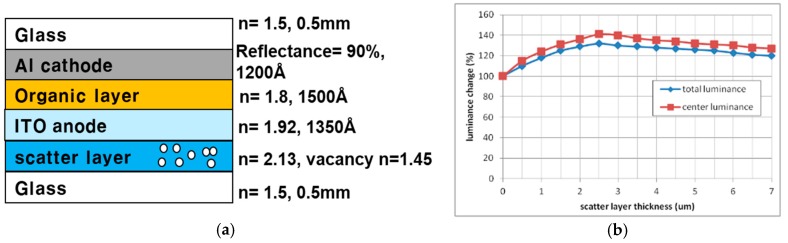
Simulated luminance change of the OLED device with the implemented YSZ scattering layer: (**a**) Schematic diagram and parameters of the OLED device; (**b**) Simulated total luminance and center luminance to normal direction as a function of the thickness of the scattering layer.

Table 3 shows the relative luminance of OLED devices with the implemented YSZ nanoparticle scattering layer compared to the control device without the scattering layer. In the presence of the scattering layer, the total luminance and luminance in normal direction of the OLED device enhanced, compared to the reference device without the scattering layer. At the thickness of scattering layer, 2.5 μm, a 29% enhancement of total luminance and a 38% enhancement of luminance in normal direction were achieved. As the result, the optimal thickness of the scattering layer was observed at 2.5 μm. Moreover, the luminance enhancement is not proportional to the increase of the scatter effect. As the thickness of scattering layer was increased, the luminance decreased. This means that the transmittance of the thick scattering layer lowers as the thickness increases. To analyze optimal thickness of the scattering layer implemented in the OLED device, the luminance change was simulated using a geometrical optics tracing method. The LightTools program (Synopsys, Mountain View, CA, USA) was used for the simulation. As Figure 5a shows, the simplified device structure used for the simulation also approximated constant values of parameters such as the refractive index and the thickness of each layer. The maximum luminance enhancement was calculated as 32% enhancement at a thickness of 2.5 μm, in good agreement with the experimental result. Moreover, the maximum luminance enhancement in normal direction was calculated as 42% at the center of the device. Figure 5 shows the simulation result of luminance change in the OLED device.

**Figure 6:**
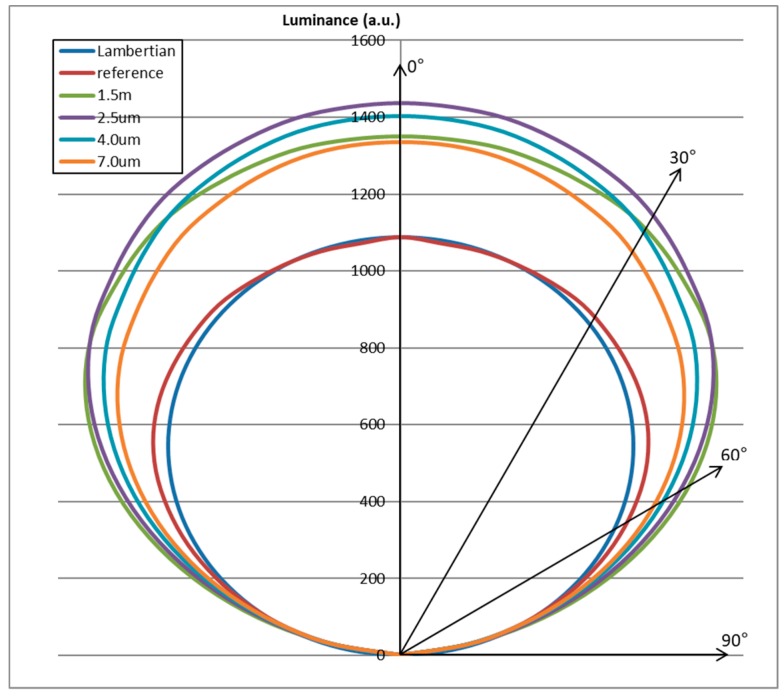
Relative angular luminance distribution of OLED devices with the implemented YSZ nanoparticle scattering layer versus the thickness of scattering layers, compared to the reference device and Lambertian distribution. The Lambertian distribution is plotted using scale-up to the same value of reference device at 0 degree for relative comparision.

**Figure 7:**
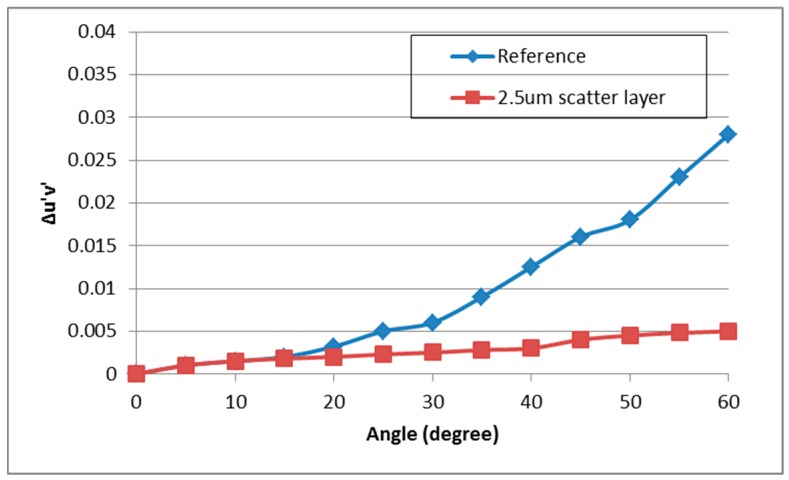
Change of color coordinates (Δu’v’) of the OLED device with the implemented 2.5 μm thick YSZ nanoparticle scattering layer versus the viewing angle.

Figure 6 illustrates the relative angular luminance distribution of the OLED device with the implemented YSZ nanoparticle scattering layer as a function of thickness of the scattering layers. Compared to Lambertian distribution, for the reference device it was observed that the luminance at high angles was larger than that at normal direction. The OLED devices with implemented scattering layers showed enhanced luminance distribution at all the angles. Moreover, at the scattering layer thickness of 2.5 μm, the most enhanced luminance was achieved. Consequently, the YSZ nanoparticle scattering layer enhanced the light extraction efficiency to all angular directions by the optimized scatter effect. Moreover, enhanced light extraction at high viewing angles improved the angular color change of the OLED device. As Figure 7 shows, the white angular dependency (WAD), which represents color change by angular viewing direction, decreased significantly. The color coordinate change (Δu’v’) of the reference device at 60° was 0.028, while that of the OLED device with the 2.5 μm thick scattering layer was 0.005, which is an 82% decrease of color change. Hence, due to the scatter effect of the YSZ nanoparticle scattering layer, we achieved not only enhanced light extraction efficiency but also improved angular color change of the OLED device.

## 4. Conclusions

The implemented YSZ nanoparticle material and planarization material endured, without deformation, the subsequent thermal annealing process at the temperature ranged to 580 °C. Highly efficient OLED devices can be manufactured using the conventional display manufacturing process by implementing YSZ nanoparticles. A 38% enhancement in luminance of the OLED device was achieved by the scattering layer with a thickness of 2.5 μm, compared to the device without the scattering layer. Moreover, the decreased angular color change was obtained by implementation of the nanoparticle scattering layer, where the white angular dependency (WAD) Δu’v’ was reduced to 0.005 at 60 degrees from normal direction. Thus, we believe that this study can provide a simple, practical, and low-cost method for improving the performance of OLED display products.

## Figures and Tables

**Table 1:** Transmittance and haze of the TiO_2_ nanoparticle scattering layer.

Layers	Total Transmittance (%)	Scattering Transmittance (%)	Haze
**Scattering layer (1.5 μm)**	70.18	57.54	82%
**Scattering layer (1.5 μm)** **/Planarization layer (1 μm)**	68.28	37.55	55%

**Table 2:** Transmittance and haze of the YSZ nanoparticle scattering layer.

Thickness of Scattering Layer (μm)	Thickness of Planarization Layer (μm)	Total Transmittance (%)	Scattering Transmittance (%)	Haze
1.5	1.0	78.43	19.13	24%
2.5	1.0	74.63	20.06	27%
4.0	1.0	65.84	25.47	39%
7.0	1.0	58.43	32.15	55%

**Table 3:** Ratio of the luminance of OLED devices with the YSZ scattering layer to the luminance of the reference OLED without the scattering layer, for various layer thickness.

Thickness of Scattering Layer (μm)	Changed Ratio of Total Luminance	Changed Ratio of Normal Direction Luminance
0 (without scattering layer)	100%	100%
1.5	123%	131%
2.5	129%	138%
4.0	126%	135%
7.0	119%	128%
